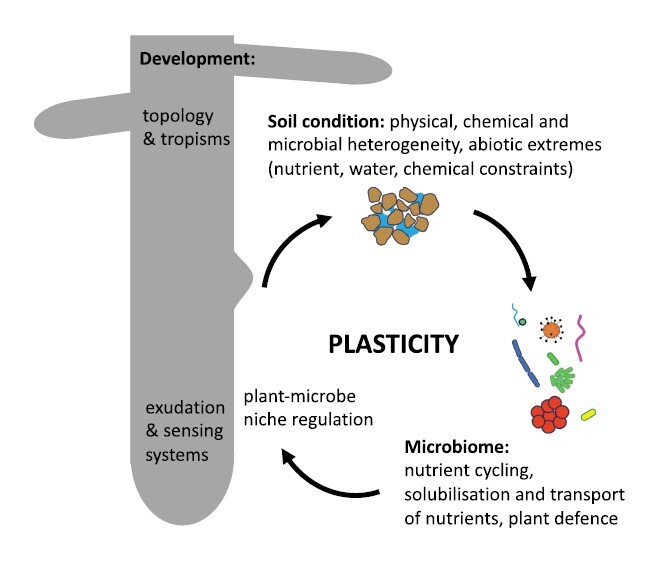# Root architecture and rhizosphere–microbe interactions

**DOI:** 10.1093/jxb/erad488

**Published:** 2024-01-08

**Authors:** Miriam L Gifford, Guohua Xu, Lionel X Dupuy, Kris Vissenberg, Greg Rebetzke

**Affiliations:** School of Life Sciences, The University of Warwick, Coventry, UK; National Key Laboratory of Crop Genetics & Germplasm Enhancement and Utilization, Nanjing Agricultural University, Nanjing 210095, China; Department of Conservation of Natural Resources, Neiker, Derio, Spain; Ikerbasque, Basque Foundation for Science, Bilbao, Spain; Integrated Molecular Plant Physiology Research, Department of Biology, University of Antwerp, Antwerp, Belgium; Plant Biochemistry and Biotechnology Lab, Department of Agriculture, Hellenic Mediterranean University, Stavromenos PC 71410, Heraklion, Crete, Greece; CSIRO Agriculture and Food, PO Box 1700, Canberra ACT 2601, Australia

**Keywords:** Abiotic stress, CEP peptides, gravitropism, lateral root, rhizosphere, root architecture, root development, root–microbe interaction, root–soil interaction

## Abstract

Plant roots fulfil crucial tasks during a plant’s life. As roots encounter very diverse conditions while exploring the soil for resources, their growth and development must be responsive to changes in the rhizosphere, resulting in root architectures that are tailor-made for all prevailing circumstances. Using multi-disciplinary approaches, we are gaining more intricate insights into the regulatory mechanisms directing root system architecture. This Special Issue provides insights into our advancement of knowledge on different aspects of root development and identifies opportunities for future research.

The influence of the root system on soils rarely extends beyond a few millimetres from the epidermis (Kuzyakov and Razavi, 2019), and consequently root system architecture (RSA) has often been linked to the ability of a plant to acquire soil resources (Fitter, 1986; Lynch, 1995). Ultimately, the spatial distribution of roots in soil determines the overall volume explored by the plant, the fraction of the soil occupied by the roots, or the surface through which water and minerals transit. The topology of the RSA and the conductivity of root tissues together determine the resistance to movement of water and nutrients to the shoot. Using architectural representation of the root system, one can therefore propose ideotypes from the knowledge of how resources are distributed in soil such as the ‘steep, cheap and deep’ ideotypes for optimal water uptake efficiency (Lynch, 2013; Uga *et al.*, 2013). Therefore, in our quest for enhancing crop productivity with the increasing threat from climate change, fundamental knowledge on root development and interactions with the rhizosphere is needed to tackle significant 21st century challenges (Lynch, 2022). While foraging for heterogeneously distributed resources such as water and nutrients, the root system must adapt to highly diverse soil conditions and to biota that are present in its rhizosphere. As a result, the integration of all these environmental signals shapes the root system throughout its lifetime and adapts its architecture for optimal resource capture (Morris *et al.*, 2017). Through the development of powerful ‘omics techniques, visualization methods, and mathematical models, and in combining different experimental approaches, we are increasing our understanding of the mechanisms underlying root growth and responses to diverse environmental signals. This Special Issue highlights recent understanding on root system development and plasticity of root architecture towards abiotic stressors, and covers recent breakthroughs in root–rhizosphere and root–microbe interactions.

Our understanding of the functioning of root systems is changing rapidly. Studies are now consistently pointing to the effect of root phenotypic plasticity on fitness in a natural environment (Keser *et al.*, 2014; Hiatt and Flory, 2020), and it has even been proposed that root plasticity should become a breeding target (Schneider and Lynch, 2020). Therefore, fundamental studies identifying the regulatory pathways for lateral root development are becoming critical in developing a mechanistic understanding of root plasticity (Banda *et al.*, 2019), since it is the process controlling topology of the root system. In this issue, Dwivedi *et al.* (2024) used a genetic mapping approach to identify *CaWIP2*, a C2H2 zinc finger transcription factor gene contributing to the formation of lateral roots in chickpea. Like its orthologues in Arabidopsis, *CaWIP2* is preferentially expressed in the root apical meristem and lateral root primordia. *CaWIP2* is able to rescue root development of the *wip2/4/5* Arabidopsis mutant, suggesting that *CaWIP2* is a positive regulator of lateral root development in plants (Dwivedi *et al.*, 2024). In addition to the length and number of lateral roots, root angle also determines their ability to explore larger soil volumes. Chapman *et al.* (2024) report that C-terminally encoded peptide (CEP) and cytokinin pathways intersect to impact the initial angle of the emerging lateral root. They find that CEP-induced promotion of a shallower lateral root growth angle relies on the action of cytokinin biosynthesis, transport, and perception, while a fully functional CEP pathway is also critical for achieving the regulation by cytokinin of lateral root growth angle. This intersection between CEP and cytokinin pathways provides an insight into the determination of spatial distribution patterns of plant root systems (Chapman *et al.*, 2024). Additionally, Porat *et al.* (2024) developed a mathematical model to describe the dynamics of root gravitropism, providing a quantitative understanding of this root tropism. Taleski *et al.* (2024) review the widespread role of the CEPs, which are a large class of peptides with a range of well-conserved roles in regulating nitrogen-related development across species and can be thought of as acting as hormones. The complexity of expression and processing of CEP gene products into mature CEP peptides potentially enables their regulation. As well as regulating local and long-range nitrogen signalling to influence RSA within non-legumes such as Arabidopsis, CEPs have been known for some time to be key in regulating (and probably co-regulating) nodulation with lateral root development in legumes such as Medicago. This review outlines key recent work that helps to link CEP activity with development, including other key hormones such as auxin, and also the extent to which they signal external (environmental) versus internal (derived from nodulation) nitrogen levels (Taleski *et al.*, 2024).

It is also becoming increasingly clear that rhizosphere microbiology is critical to understand how root systems function. Rhizodeposits (Pausch and Kuzyakov, 2022) are exploited by the soil microorganisms affecting nutrient cycling (e.g. Henneron *et al.*, 2020) or contributing to plant defence (e.g. Yuan *et al.* 2018). Furthermore, rhizosphere microbes also have a formidable ability to manipulate the development of the root system itself. This has been well documented in the case of nitrogen fixers (Hawkins and Oresnik, 2022), but root–microbe interactions are now also being linked to modifications of growth rate, apoplastic barriers, and lateral root initiation (Salas-González *et al.*, 2021; Chiu *et al.*, 2022; Gonin *et al.*, 2023). In this Special Issue, Pathak *et al.* (2024) review the roles of nitric oxide (NO) and phytoglobins in symbiotic interactions, with a particular focus on the more well-studied system of rhizobia–legume symbiosis. The review reveals the complexity of signal interplay at the membranes of the plant and bacteroid in the nodule, showing how enzymatic activity on each leads to exchange of forms of NO across the peribacteroid space. Phytoglobins are one class of the very large group of haemoglobins, of which the classification linked to their function is complex and evolving. Regulatory roles for phytoglobins are being elucidated not just in symbiosis and legumes but also in many other growth and developmental processes outside of these plants.

Small molecule pathways involved in signalling are being identified with increasing resolution, leading to findings that develop our mechanistic understanding of interactions between organisms. For example, in novel research, which is discussed in this Special Issue (Dong *et al.*, 2024), by using an aequorin-based calcium indicator, Binci *et al.* (2024) quantified signalling related to the key secondary messenger Ca^2+^ in the cytoplasm and nucleus when *Lotus japonicus* plants were treated with different chitin-derived fungal elicitors. This has helped elucidate the details of the role it plays in modulating symbiotic outcome, delineating the dynamics of Ca^2+^ pool location in the cytoplasm and nucleus, as well as timing, but also determining the extent of requirement for the common symbiotic signalling pathway for responding to fungal signals.

In the Expert View by Galindo-Castaneda *et al.* (2024), a holistic view of plant root–microbe interactions is proposed, examining our understanding of the spatial location of rhizobial and mycorrhizal interactions and how this is driven by exudates and nutrient limitation as key factors, within the context of RSA. Exciting progress has also been made in high-resolution imaging which allows unprecedented observation of root responses to abiotic and also biotic factors. Such techniques considerably expand our understanding of the phenotypic impact of roots on microbes, and vice versa. Galindo-Castaneda *et al.* highlight a number of recent findings that link rhizosphere composition or processes to root developmental stage, age, or type, with consequences back to/on the rhizosphere. The authors underscore the importance of including evaluation of RSA effects/context and of working in systems that enable effects of the heterogenous real soil environment to be evaluated, using soil vertical—and also horizontal—gradients. Doing this within studies that examine the root microbiome and microbial processes would bring us closer to understanding the true root system.

Our current understanding of plant–microbe interactions on shaping RSA with relevance for applications in agricultural production is discussed in Li *et al.* (2024). Their review synthesizes the current knowledge of plant–microbe interactions at a variety of molecular levels, and links microbial activity to plant molecular pathways via the natural products exuded by microbes that influence the root directly (such as hormone-like molecules) or indirectly, influencing plant gene expression. This synthesis of knowledge will help in pinpointing targets for plant breeding, but also in developing use and synthesis of microbial bioactive products.

Abiotic constraints limiting root elongation are well established to restrict root growth and function. However, there is limited understanding of the molecular and physiological basis that might allow for agronomic and genetic manipulation in development of more resilient crops when challenged by abiotic constraints. In the Expert View of Pandey and Bennett (2024), the release of ethylene from root tips and the restricted diffusion of ethylene in compacted soils are described, and both anatomical features and target genes towards genetic manipulation in breeding are identified. Ethylene together with hypoxia are also key in limiting the growth of roots in waterlogged soils characterized by hypoxic conditions. Understanding of plant responses to hypoxia has largely focused on flood-tolerant crops and other plant species. Daniel and Hartman (2024) review how changes in root growth and overall RSA in avoiding and/or enduring waterlogging by non-flood-adapted species may provide new physiological and molecular insights in breeding of waterlogging-susceptible crops. The importance of tolerance to reduced soil water is explored in the context of root–soil hydraulic conductivity. In a comparison between two closely related yet morphologically contrasting species, Hostetler *et al.* (2024) review how differential root architectural responses to changes in soil moisture, salinity, and phosphorus may lead to the identification of new root ideotypes for wider adaptation to climate change and translational opportunities to select more resilient crops.

Soil modifications brought about by biological activity in the rhizosphere also have knock-on effects on the properties of soil and the physical processes of transport of water, dissolved nutrients, and microorganisms. It has long been known from soil hydrologists that vegetation improves the infiltration of water through mechanisms such as preferential flow (Bouma, 1981; Flury *et al.*, 1994) and, consequently, this can reduce runoff and soil erosion (Zuazo and Pleguezuelo, 2009; Cerdan *et al.*, 2010). Yet, detailed analysis of the physical properties of root exudates are only beginning to reveal the properties of rhizosphere soils (Naveed *et al.*, 2019). Measurements of flows in the rhizosphere are technically challenging, but mathematical models predict that preferential flows of soil water induced by root systems may be an efficient mechanism to distribute water in soil and resist drought (Mair *et al.*, 2023). In their Expert View, Afforfit *et al.* (2024) provide an understanding of how plants adapt to the rhizosphere in changing RSA and interactions with the soil microbiome. New opportunities towards breeding improved root–soil interface adaptation provide promising directions for further advances in this field.

## Conclusion

The development of RSA results from genetically encoded developmental mechanisms that are modulated by a multitude of signals from the surrounding environment (Box 1). The intricacy of the environmental signals, the difficulty of observing roots/soil *in situ*, species composition within root microbiomes, and the overlap between pathways shaping root architecture according to disparate signals and organisms are amongst the complexities that are major limitations to our understanding of RSA. The studies published in this Special Issue show that breakthroughs are nevertheless possible, and that these are often the results of multidisciplinary studies where both the root system and the environment are treated as interacting factors.

Box 1. **The plasticity of the root system in response to environmental conditions**

Although the development of the root is genetically regulated, parameters such as elongation rate, root diameter, cell length, or branching rates are known to vary significantly in response to changes in soil conditions. Many of the responses of the root system remain poorly understood. By viewing the rhizosphere as a system where plant and microbial molecular processes interact with the physical environment, the latest research in this field is gradually uncovering the mechanisms underlying root system plasticity.